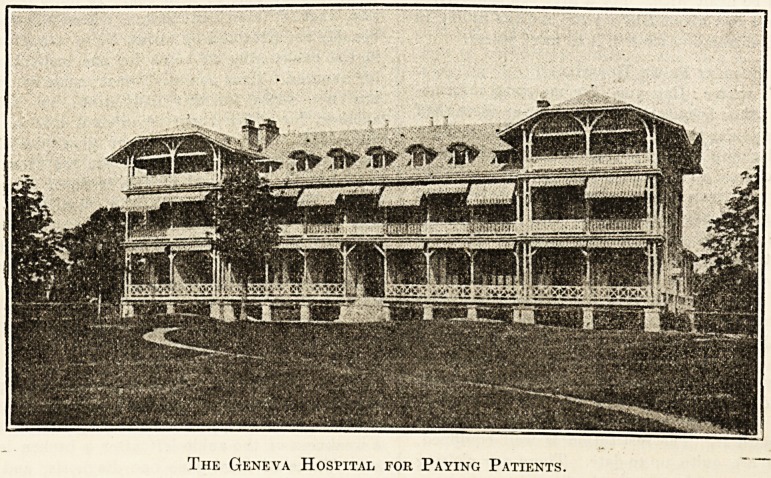# A Swiss Hospital for Paying Patients

**Published:** 1911-12-23

**Authors:** Conrad W. Thies

**Affiliations:** late Secretary Royal Free Hospital.


### A SWISS HOSPITAL FOR PAYING PATIENTS.

By CONRAD W. THIES, late Secretary Royal Free Hospital.

In Great Britain, especially in London and the large provincial cities, the poorer classes in case of sickness enjoy the great privilege of free treatment, either in the voluntary hospitals or in the Poor Law Infirmaries; whereas members of the middle class must be nursed in their own homes or in private institutions where the charges are, generally speaking, very high.

After visiting the large cantonal hospitals in Geneva and Lausanne, I was much interested in visiting a private clinique, or hospital for paying patients, in Geneva. The General Clinique of Florissant was founded a few years since, and is carried on by a small public company for the purpose of affording the best medical and surgical treatment to those who are not considered eligible for admission to the public hospitals. It must be borne in mind that there are no really free patients in any of the public hospitals of Switzerland; every patient is paid for, either by the public authority of the district where the patient resides or by his friends.

The hospital is situated on the outskirts of Geneva; it was specially built for the purpose on an excellent site in the midst of beautiful gardens.

The principal rooms for patients face to the south, and command extensive views of the valley of the river Arve and of the Salève mountains. The institution is administered by a board of directors—one-half of whom are medical practitioners and one-half laymen. The management is entrusted to a lady superintendent, who has had practical experience in a public hospital and is assisted by a competent staff of trained nurses and domestic assistants. The hospital is licensed and systematically inspected by the public health authorities of the canton. In addition to the rooms reserved for the staff, accommodation is provided for forty patients. These are of two classes, of whom the first class pay from 12 to 15 francs, and the second class 7 to 10 francs per day. These charges cover the cost of board, lodging, and the usual nursing attendance; but baths, massage, medicines, dressings, etc., are extras. For the use of the operating room first-class patients pay 30 francs (additional), and second-class 15 francs.

For the first-class patients there are fifteen separate rooms, each provided with a spacious balcony which can be screened off by blinds. The second-class patients are accommodated in rooms containing two or four beds. On each floor there is an adequate provision of bath-rooms and lavatories, and on the basement floor a very complete installation of baths and electrical appliances of all kinds. All the rooms are most comfortably furnished, with every kind of appliance for the use of the patients, while the open-air balconies are supplied with lounges and easy chairs. The walls of all the rooms are enamelled and decorated in oil paint, so that they can be readily washed and disinfected. The two operation-rooms are planned and equipped on the most modern principles. No medical officer is attached to the hospital; the patients are attended by their own physician or surgeon, whose fees they pay in the ordinary manner. The only stipulation made by the directors is that the doctor must be a recognised practitioner in the canton of Geneva. The form of admission to the hospital must in all cases be signed by a medical practitioner.

The Geneva Hospital for Paying Patients.

All kinds of medical and surgical cases are received, except infectious cases; special attention is given to patients suffering from nervous affections and those requiring isolation and repose. The suite of rooms for maternity cases is entirely separated from the rest of the hospital. I cannot conclude these remarks without expressing my admiration of all the arrangements of this excellent hospital, which exactly meets the need of a class of patients who are usually precluded from the benefit of treatment in cases of serious illness in a thoroughly up-to-date modern hospital. The Home Hospitals Association in Fitzroy Square, which owes its foundation to Sir Henry Burdett, is perhaps the nearest counterpart in London of this Swiss hospital.

## Figures and Tables

**Figure f1:**